# The difference in knowledge and attitudes of using mobile health applications between actual user and non-user among adults aged 50 and older

**DOI:** 10.1371/journal.pone.0241350

**Published:** 2020-10-27

**Authors:** Mangyeong Lee, Danbee Kang, Junghee Yoon, Sungkeun Shim, Im-Ryung Kim, Dongryul Oh, Soo-Yong Shin, Bradford W. Hesse, Juhee Cho

**Affiliations:** 1 Department of Digital Health, SAIHST, Sungkyunkwan University, Seoul, South Korea; 2 Center for Clinical Epidemiology, Samsung Medical Center, Sungkyunkwan University School of Medicine, Seoul, South Korea; 3 Department of Clinical Research Design and Evaluation, SAIHST, Sungkyunkwan University, Seoul, South Korea; 4 Cancer Education Center, Samsung Comprehensive Cancer Center, Samsung Medical Center, Sungkyunkwan University School of Medicine, Seoul, South Korea; 5 Department of Radiation Oncology, Samsung Medical Center, Sungkyunkwan University School of Medicine, Seoul, South Korea; 6 Big Data Research Center, Samsung Medical Center, Sungkyunkwan University School of Medicine, Seoul, South Korea; 7 National Cancer Institute, National Institutes of Health, Bethesda, MD, United States of America; 8 Departments of Health, Behavior and Society and Epidemiology, Johns Hopkins Bloomberg School of Public Health, Baltimore, MD, United States of America; Edinburgh Napier University, UNITED KINGDOM

## Abstract

**Background:**

Despite the great benefits of mobile health applications (mHAs) in managing non-communicable diseases (NCDs) internationally, studies have documented general challenges to broad adoption of mHAs among older age groups. By focusing on broad adoption, these studies have been limited in their evaluation of adults aged 50 and older who have high risk of NCDs and can benefit the most from the functionalities provided by mHAs.

**Objective:**

This study aims to evaluate the knowledge, self-confidence, perceived benefits, and barriers of using mHAs depending on experience with mHAs among adults aged 50 and older. Furthermore, we aim to identify the factors associated with the actual use of mHAs.

**Methods:**

We conducted a cross-sectional survey at a single tertiary hospital in Seoul, Korea, between May 1 and May 31, 2018. Of the 625 participants who were contacted, 323 participants were granted full inclusion to the study. We compared demographics, knowledge, self-confidence, and perceived benefits and barriers by experience with using mHAs, then performed logistic regression to identify the factors associated with mHA use.

**Results:**

Among the participants, 64.1% (N = 207) had experience using mHAs. Those in the experienced group were more likely to have more than college education (55.1% vs. 27.5%, P < 0.001) and to report a higher monthly income (≥ $7,000, 22.7% vs. 18.1%, P = 0.05) than their less-experienced counterparts. Although the experienced group was more likely to have higher self-confidence in using mHAs, about half of the study participants, including people with experience using mHAs, did not have appropriate knowledge of mobile technology. With adjusted logistic model, higher educated (adjusted PR (aPR) = 1.53, 95% CI, 1.26–1.80), higher perceived benefits of mHAs (aPR = 1.43, 95% CI, 1.04–1.83), and higher self-confidence using mHAs (aPR = 1.41, 95% CI, 1.12–1.70) were significant factors associated with mHA use.

**Conclusions:**

The use of mHAs among adults aged 50 and older is becoming more common globally; nevertheless, there are still people unable to use mHAs properly because of lack of experience and knowledge. Strategies are needed to encourage the reliable usage of mHAs among those who may need it the most by improving self-confidence and better articulating benefits.

## Introduction

As society ages and life expectancy increases, it has become a global public health priority to prevent and manage non-communicable diseases (NCDs) [1, 2]. Estimates suggest healthcare expenditures to manage NCDs could reach nearly $47 trillion by 2030 [3]. The average age of major chronic NCDs (cardiovascular disease, diabetes, or cancer) is around 50s and 60s [4, 5]. According to the data from the National Health Interview Survey, 2012, six out of ten adults aged 45–64 adults have at least one and more NCDs [6]. The United States Center for Disease Control and Prevention (CDC) reported that the number of adults aged 45–64 with multiple chronic conditions increased between 2000 and 2010 [7]. Considering the social and economic burden of NCD, mobile health applications (mHAs) can be an attractive health care system option to support patients and to encourage the adoption of healthy lifestyles and NCD self-management [8, 9]. Over the past decade, many mHA clinical trials have demonstrated they can improve symptoms and quality of life, stabilize metabolic function, and reduce hospitalization or death related to NCDs [10–18].

Despite the great benefits of mHAs, challenges remain to their adoption and actual use [19–22]. An estimated 325,000 mHAs have been available in major app stores since 2017, but previous studies report that adoption and continued use remain low [23–27]. According to a 2014 market report conducted in 27 countries, only 1.2% (1.6M/133M) of diabetic patients used a smartphone app to manage their disease [28]. This appears to be particularly true among adults aged 50 and older at high risk of NCDs [29]. In fact, according to the National Health and Nutrition Examination Survey, mean age at diagnosis of type 2 diabetes and hypertension are 51.1 and 47.8, respectively [30]. These adults might experience more barriers to mHA use as they are more familiar with an analog lifestyle and may feel less attracted to advanced digital technology [22, 31, 32]. Moreover, as aging decreases visual and cognitive function, older adults would have more difficulties using mHAs on the small screen of a smartphone [24]. Furthermore, previous studies found that old adults experienced low self-efficacy in using mHAs due to lack of technical proficiency, and they had low intention to use the technology [19, 24, 33]. Nevertheless, many within these same age groups have expressed a strong interest in using mobile solutions to manage their conditions; they have simply reported obstacles and barriers in usage. Because of the medical value of preemptive, mHA-enhanced care–and the fact that older populations have expressed an interest in using mobile solutions–researchers have emphasized the importance of developing strategies to encourage usage [24, 34, 35].

According to two nationwide surveys in the United States and Germany in 2015, mHA users were more likely to be younger, have higher education, and have chronic conditions [36, 37]. In other studies, perceived ease of use and usefulness were associated with intention to use mHAs [20, 26]. Some studies reported that, in general, health literacy and self-confidence were associated with mHA adoption [36, 38, 39]. What has been lacking, however, has been an explicit focus on the population which also includes adults in their 50s; that is, the very populations that would experience the greatest benefit from mHAs. Few quantitative studies assessed knowledge, beliefs, and attitudes about using mHAs among the older population, taking adults in their 50s into account. [20, 36]. An understanding of knowledge, beliefs, and attitudes within this population should help add much-needed specificity to enhance the general technology adoption framework utilized in previous research. This study, then, aims to evaluate the knowledge, self-confidence, perceived benefits, and barriers to using mHAs among adults aged 50 and older, while taking into account previous experience. Furthermore, we aim to identify the factors associated with the actual use of mHAs in the context of controlling and managing NCDs.

## Methods

### Participants

**Fig 1:**
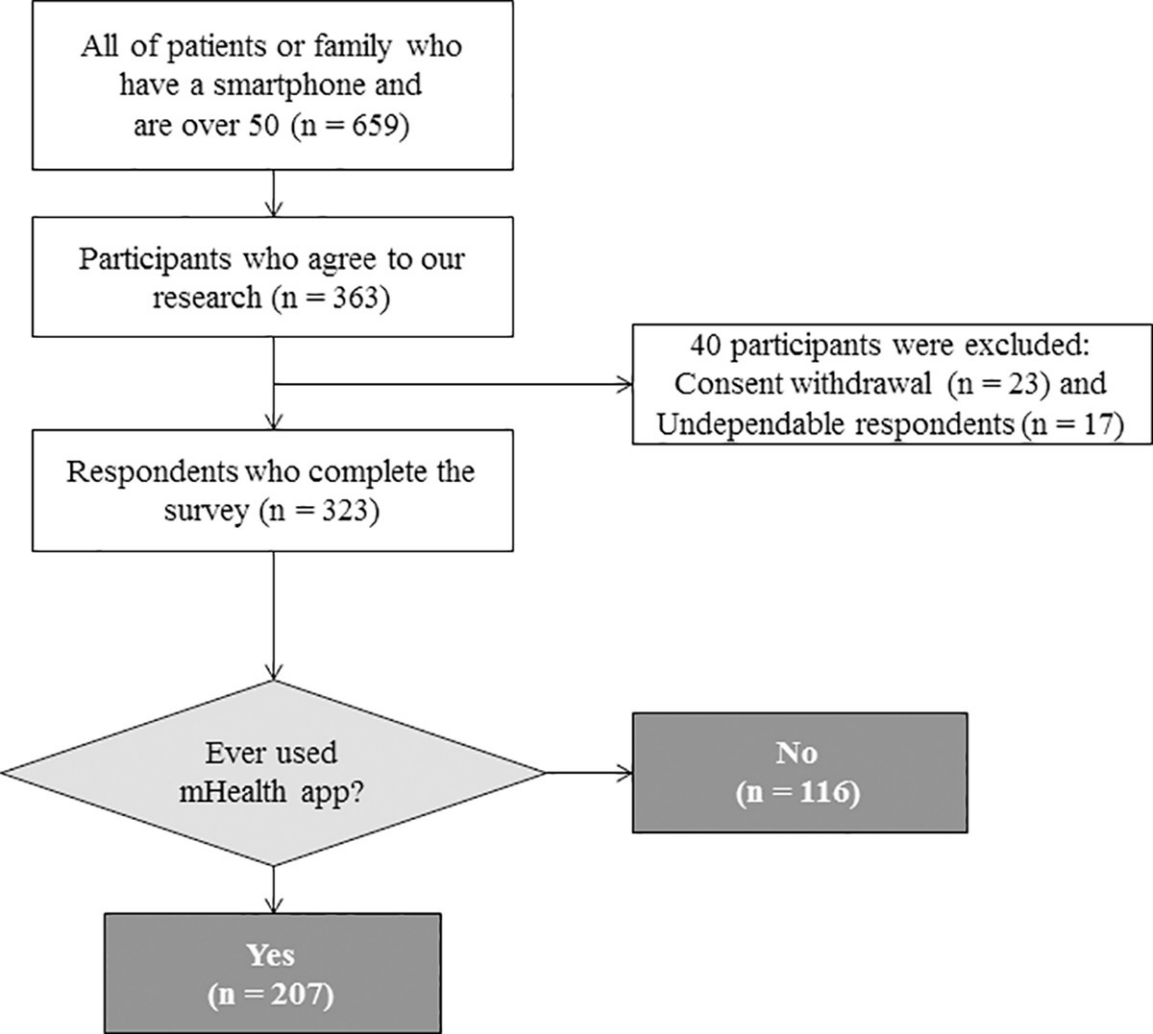
Flowchart of the study and operational definition of subjects.

We conducted a cross-sectional survey at the Samsung Medical Center in Seoul, Korea, between May 1 and May 31, 2018. We recruited adults aged 50 and older who currently used a smartphone. At an outpatient clinic and inpatient room, trained researchers explained our research to patients, caregivers, or visitors, then those who wanted to participate in the survey were asked to mark ‘yes’ in the checkbox of the informed consent at the top of the questionnaire. For almost 15 to 20 minutes, participants were asked to answer the survey questionnaire, then we gave a gift worth under 5,000 KRW (5 dollars) to those who completed the survey without withdrawal. Of the 625 patients who were contacted, 363 (58.1%) agreed to participate in the study. After excluding participants whose surveys were incomplete (*N* = 40), 323 participants were included in this study (Fig 1). The study was approved by the Institutional Review Board of the Samsung Medical Center (SMC 2017-08-121).

### Measurement

We developed a questionnaire based on a literature review, qualitative interviews, and expert opinion. First, we reviewed previous studies that examined factors associated with mHA adoption and developed a conceptual framework using the Health Belief Model [20, 24, 26, 33–35]. We also reviewed previous national surveys that examined similar behaviors [40]. Next, we conducted semi-structured interviews with 30 people (including patients with NCDs) to assess knowledge of mHAs as well as perceived benefits and barriers to adoption. Based on the results of qualitative interviews, we developed a 22-question survey to assess knowledge (n = 5), self-confidence (n = 3), perceived benefits (n = 6), and barriers (n = 8) to mHA use (**S1 File**). Content validity was confirmed by the expert group, which consisted of 2 behavior scientists, 2 nurses, 2 clinicians, and 2 digital health experts.

We conducted a pilot study in April 2018 at Samsung Comprehensive Cancer Center. We asked 10 cancer patients who meet our inclusion criteria to participate in the study, all of whom had been introduced to mHA upon diagnosis. Once they agreed to participate, they completed the questionnaire. Following survey completion, we asked participants about appropriateness of the length, logic, and wording of the questionnaire. All participants indicated the questionnaire was not difficult to complete, but we found the 2 knowledge assessment questions to be too easy and lacking discriminant value. We decided to exclude these 2 mHA knowledge questions, resulting in 20 questions total. To assess their experience using mHAs, we asked participants whether they had used mHAs in last year (dichotomous questions: yes or no). To identify factors associated with mHA use, we asked about their knowledge, self-confidence, and perceived barriers and benefits. We measured the knowledge needed to use mobile health solutions by asking them to choose the correct meanings of the WiFi, GPS, and Bluetooth signs in the smartphone. As these icons are standardized regardless of device type, this was useful to discriminate knowledge among participants. Self-confidence was assessed using a 4-point Likert scale (1 = *strongly disagree*, 2 = *disagree*, 3 = *agree*, 4 = *strongly agree*). Participants were asked to quantify their confidence in managing their health through mHAs, including confidence in using a smartphone. We also measured perceived barriers and benefits to using mHAs. Perceived barriers consisted of physical limitation (*n* = 2), cost and privacy concerns (*n* = 2), lack of support (*n* = 2), and misunderstandings about using mobile health functionality (*n* = 2). Perceived benefits consisted of minimizing time and space constraints of conventional healthcare (*n* = 3) and increased accessibility to manage health (*n* = 3). Participants answered how far they agreed with the statements on a 4-point Likert scale (1 = *strongly disagree*, 2 = *disagree*, 3 = *agree*, 4 = *strongly agree*). Health literacy was evaluated with the Newest Vital Sign (NVS), which measures participant health literacy by asking 6 questions regarding an ice cream container nutrition label [41]. In this study, we used the Korean version of NVS, which is validated and used in many previous studies [42–44].

Lastly, we asked participants their sociodemographic details including gender, age, educational level, household income, living area, health literacy, and chronic comorbidities, including hyperlipidemia, hypertension, stroke, myocardial infarction, angina pectoris, and atherosclerosis.

### Statistical analysis

We separated the participants into two groups (the experienced and the control) by experience using mHAs. For analysis of confidence in using mHAs and perceived benefits and barriers, the participants who responded “strongly agrees” or “agrees” were grouped into the “yes” category, while the participants responding “disagrees” or “strongly disagrees” were grouped into the “no” category. We then performed descriptive analysis to compare respondents’ characteristics, knowledge, self-confidence, and perceived barriers and benefits between the two groups. We used *χ*^2^-tests and *t*-tests to determine the statistical significance of the differences between two groups.

We used univariable and multivariable logistic regression models to identify factors associated with mHA use. We calculated prevalence ratios using the predicted proportion from logistic regression. For the analysis, knowledge and health literacy were dichotomized into ‘limited’ (knowledge score of 0–1 and NVS score of 0–3) and ‘adequate’ (knowledge score of 2–3 and NVS score of 4–6). Self-confidence in using mHAs, and perceived barriers and benefits were dichotomized into ‘high’ and ‘low’ at their mean of medians. For the multivariable analysis, we adjusted for gender (male vs. female), age (< 60 years vs. ≥ 60 years), education (≤ High school vs. ≥ College), and comorbidity, all well-known factors associated with mHA use. All statistical analyses were performed using STATA 14.0 (Stata Corp LP, College Station, Texas, USA), and statistical significance was defined as 95% confidence interval and *P* < 0.05.

## Results

### The characteristics of participants

The mean age of the study participants was 60.9 (*SD* = 6.43), and 62.9% of the participants were female. Among the 323 participants, 64.1% (*N* = 207) had experience using mHAs in the last year. Compared to the control group, the experienced group were more likely to have more than college education (55.1% vs. 27.5%, *P* < 0.001), reported a higher monthly income (≥ $7,000, 22.7% vs. 18.1%, *P* = 0.05), and tended to be diagnosed with cancer (45.3% vs. 31.3%, *P* = 0.03) (**Table 1**).

**Table 1:** Participant characteristics by experience using mHAs.

	Overall (*N* = 323) *n* (%)	Experience using mHAs		*P*-value
		Yes (*n* = 207) *n* (%)	No (*n* = 116) *n* (%)	
**Gender**				0.97
Male	123 (38.1)	79 (38.2)	44 (37.9)	
Female	200 (62.9)	128 (61.8)	72 (62.1)	
**Age (years), mean (SD)**	60.9 (6.43)	60.5 (6.14)	61.7(6.89)	0.10
**Age category**				0.43
50s	149 (46.1)	101 (48.8)	48 (41.4)	
60s	132 (40.9)	81 (39.1)	51 (44.0)	
≥ 70s	42 (13.0)	25 (12.1)	17 (14.7)	
**Education**				< 0.001
≤ High school	172 (54.1)	90 (43.5)	82 (70.7)	
≥ College	146 (45.9)	114 (55.1)	32 (27.6)	
**Occupational Status**				0.32
Employed	164 (48.9)	109 (52.7)	55 (47.4)	
Unemployed	157 (51.1)	96 (46.4)	61 (52.6)	
**Monthly Household income**				0.05
< $3,000	105 (33.7)	57 (28.6)	48 (42.5)	
$3,000–$6,999	139 (44.5)	95 (47.7)	44 (38.9)	
≥ $7,000	68 (21.8)	47 (23.6)	21 (18.6)	
**Living Area**				0.09
Metropolitan city	140 (43.3)	97 (46.9)	43 (37.1)	
Non-metropolitan city	183 (56.7)	110 (53.1)	73 (62.9)	
**Health literacy**^**a**^				0.08
Adequate	67 (20.7)	49 (23.7)	18 (15.5)	
Limited	256 (79.3)	158 (76.3)	98 (84.5)	
**Chronic comorbidity**^**b**^	250 (77.4)	167 (80.7)	83 (71.6)	0.06
Cancer	128 (51.2)	92 (45.3)	36 (31.3)	0.01
Cardiovascular disease^**c**^	123 (49.2)	79 (38.9)	44 (38.3)	0.91
Diabetes	37 (14.8)	21 (10.4)	16 (13.9)	0.35
Arthritis	34 (13.6)	17 (8.4)	17 (14.8)	0.08
Etc.^**d**^	55 (22.0)	37 (18.3)	18 (15.7)	0.55

^**a**^ Maximum score is 6 (Limited; 0–3 score, Adequate; 4–6 score).

^**b**^ Mutually inclusive.

^**c**^ Including hyperlipidemia, hypertension, stroke, myocardial infarction, angina pectoris, and atherosclerosis.

^**d**^ Including liver diseases, asthma, COPD, and benign prostatic hyperplasia.

### Knowledge of digital technology and self-confidence in using mHAs

Of the participants, 47.1%, 59.1%, and 43.3% correctly answered questions related to understanding the functionality of Wi-Fi, GPS, and Bluetooth, respectively (**Table 2**).

**Table 2:** Knowledge of digital technology and self-confidence by experience using mHAs.

	Overall (*N* = 323) *n* (%)	Experience using mHAs		*P*-value
		Yes (*n* = 207) *n* (%)	No (*n* = 116) *n* (%)	
**Understanding of current status of a smartphone**^**a**^				
Connecting to Wi-Fi	152 (47.1)	95 (45.9)	57 (49.1)	0.17
Using location service with GPS	191 (59.1)	136 (65.7)	55 (47.4)	< 0.01
Pairing other devices with Bluetooth	140 (43.3)	105 (50.7)	35 (30.2)	< 0.001
**Self-confidence in using mHAs**^**b**^				
I am confident in using a smartphone well	192 (59.4)	140 (67.6)	52 (44.8)	< 0.001
I am confident in using mHealth apps well with a smartphone.	163 (50.5)	117 (56.5)	46 (39.7)	< 0.05
If I learn, I can manage my health well with a smartphone.	269 (83.3)	185 (89.4)	84 (72.4)	< 0.001

^a^ Answered correctly to each question.

^b^ Combined ‘agree’ and ‘strongly agree’.

In terms of confidence, 59.4% and 50.5% of the participants agreed that they have confidence in using a smartphone well and using mHAs well with a smartphone, respectively. In addition, 83.3% of the participants agreed that if they learn, they can manage their health well with a smartphone.

The experienced group was more likely to have higher knowledge of GPS (65.7% *vs*. 47.4, *P* < 0.01) and Bluetooth (50.7% *vs*. 30.2%, *P* < 0.001) than the control. In addition, the experienced group was more likely to have higher self-confidence in using smart phones (67.6% *vs*. 44.8%, *P* < 0.001) and mHAs (56.5% *vs*. 39.7%, *P* < 0.05) and in managing their health with a smartphone (89.4% *vs*. 72.4, *P* < 0.001) than the control.

### Perceived benefits and barriers to using mHAs

There were many participants who agreed on the benefits of mHA use. More than half of the participants agreed that mHAs are helpful by allowing them to manage their health anywhere (79.9%) and whenever they want (76.2%) and to make consultations more easily without space restrictions (65.9%). Also 74.3%, 77.4%, and 77.4% of the participants reported that using mHAs helped them to find the health information and tailored information they needed, and that the applications were useful in helping them maintain healthy behaviors (**Table 3**).

**Table 3:** Perceived barriers and benefits depending on experience of using mHAs.

	Overall (*N* = 323) *n* (%)	Experience of using mHAs		*P*-value
		Yes (*n* = 207) *n* (%)	No (*n* = 116) *n* (%)	
**Perceive Benefits**^**a**^				
***Minimizing time and space constraints of conventional healthcare***				
The mhealth app will allow me to check my health status anytime, anywhere.	258 (79.9)	178 (86.0)	80 (69.0)	< 0.01
The mhealth app will allow me to record my health status in real time.	246 (76.2)	173 (83.6)	73 (62.9)	< 0.001
The mhealth app will allow me to receive a health consultation without having to visit a hospital.	213 (65.9)	150 (72.5)	63 (54.3)	< 0.01
***Increasing accessibility to manage health***				
The mhealth app will help me to find the health information I need.	240 (74.3)	167 (80.7)	73 (62.9)	< 0.01
The mhealth app will deliver tailored information for my health at the right time.	250 (77.4)	174 (84.1)	76 (65.5)	< 0.01
The mhealth app will help me to maintain healthy behavior.	250 (77.4)	179 (86.5)	71 (61.2)	< 0.001
**Perceived Barriers**^**b**^				
***Physical limitation***				
Even though only using a smartphone for a short time, my eyes easily tire.	151 (46.8)	92 (44.4)	59 (50.9)	0.12
The text on the smartphone screen is too small to read.	75 (23.2)	43 (20.8)	32 (27.6)	0.18
***Cost and privacy concerns***				
I am concerned about privacy violations while managing my health with a smartphone.	132 (40.9)	89 (43.0)	43 (37.1)	0.34
I think it will cost a lot of money to manage my health with a smartphone.	36 (11.2)	20 (9.7)	16 (13.8)	0.52
***Lack of support***				
There is no place where I can learn how to manage my health with a smartphone.	164 (50.8)	110 (53.1)	54 (46.6)	0.37
There is no person who can teach me how to manage my health with a smartphone.	149 (46.1)	100 (48.3)	49 (42.2)	0.58
***Misunderstanding about mHealth app***				
I don't know that mHealth app will really help me.	148 (45.8)	91 (44.6)	57 (51.4)	0.25
I think using mHealth app will increase my anxiety about health.	80 (24.8)	50 (24.5)	30 (27.3)	0.59

^a, b^ Combined ‘agree’ and ‘strongly agree’.

Of the total, 46.8% and 23.2% of the patients reported that there were physical barriers to using mHAs due to eye strain and excessively small smartphone screens, respectively. Barriers due to cost and privacy concerns were reported by 40.9% and 11.2% of the patients, respectively. In terms of lack of support, 50.8% and 46.1% of the participants felt that there was no place or person providing resources on learning how to use mHAs. In addition, 45.8% and 24.8% of the participants reported it as not helpful to managing their health and as increasing their anxiety, respectively (**Table 3**).

When we compared perceptions between the two groups, there was no statistically significant difference in perceived barriers; however, perceived benefits showed statistically significant differences between the two groups. More members of the experienced group believed that mHAs would allow them to record health data in real time (83.6% *vs*. 62.9%, *P* < 0.001), check their health status anytime and anywhere (86.0% *vs*. 69.0%, *P* < 0.01), and secure a health consultation without having to visit a hospital (72.5% *vs*. 54.3%, *P* < 0.01). Moreover, more of them believed that mHAs would make it easier to find the health information they need (80.7% *vs*. 62.9%, *P* < 0.01), receive timely, tailored information (84.1% *vs*. 65.5%, *P* < 0.01), and maintain healthy behavior (86.5% *vs*. 61.2%, *P* < 0.001).

### Factors associated with using mHAs

With the unadjusted model, higher educated (Prevalence Ratio (PR) = 3.25, 95% confidence interval (CI), 1.98–5.32), higher knowledge (PR = 2.0, 95% CI, 11.30–3.27), and higher self-confidence using mHAs (PR = 2.58, 95% CI, 1.61–4.16) were associated with using mHAs, respectively. However, health literacy was not associated with mHA use.

While perceived benefits were associated with mHA use (PR = 2.81, 95% CI, 1.62–4.86), perceived barriers were not. After adjusting for all sociodemographic factors, higher educated (adjusted PR (aPR) = 1.53, 95% CI, 1.26–1.80), higher perceived benefits of mHAs (aPR = 1.43, 95% CI, 1.04–1.83), and higher self-confidence using mHAs (aPR = 1.41, 95% CI, 1.12–1.70) were significant factors associated with mHA use (**Table 4**).

**Table 4:** Factors associated with using mHAs.

	Overall (*N* = 323)	
	Crude PR (95% CI)	Adjusted PR^a^ (95% CI)
**Gender**		
Male	*Reference*	*Reference*
Female	1.00 (0.83, 1.16)	1.12 (0.90, 1.33)
**Age**		
50s	1.14 (0.83, 1.45)	1.13 (0.81, 1.45)
60s	1.03 (0.74, 1.32)	1.04 (0.73, 1.34)
≥ 70s	*Reference*	*Reference*
**Education**		
≤ High school	*Reference*	*Reference*
≥ College	**1.49 (1.24, 1.74)**	**1.53 (1.26, 1.80)**
**Comorbidity**		
No	*Reference*	*Reference*
Yes	1.22 (0.94, 1.49)	1.31 (0.98, 1.63)
**Health literacy**^**b**^		
Limited	*Reference*	*Reference*
Adequate	1.18 (0.98, 1.39)	1.05 (0.82, 1.27)
**Knowledge of digital technology for mHAs**^**c**^		
Limited	*Reference*	*Reference*
Adequate	**1.29 (1.09, 1.53)**	1.17 (0.96, 1.39)
**Self-confidence in using mHAs**^**d**^		
Low	*Reference*	*Reference*
High	**1.43 (1.15, 1.71)**	**1.41 (1.12, 1.70)**
**Perceived Barriers**^**e**^		
High	*Reference*	*Reference*
Low	1.09 (0.89, 1.29)	1.02 (0.84, 1.21)
**Perceived Benefits**^**f**^		
Low	*Reference*	*Reference*
High	**1.54 (1.12, 1.96)**	**1.42 (1.03, 1.81)**

^a^ Adjusted for age, sex, education and comorbidity.

^b^ Limited: score of 0–1, Adequate: score of 2–3.

^c^ Limited: score of 0–3, Adequate: score of 4–6.

^d,e,f^ Dichotomized at their mean of medians.

## Discussion

In our study, about two-thirds of adults aged 50 and older had experience using mHAs; however, about half of the study participants including people with experience using mHAs exhibited a deficit in knowledge regarding the full use of mobile technologies. Participants with higher education and greater self-confidence in using mHealth functionality were more likely to be mHA users. In addition, those perceiving greater benefits were more likely to be mHA users. However, study participants seemed not to be aware of the barriers compared to benefits.

We found that over 60% of the study participants have experience with mHA use. This is higher than reported in other studies. According to the 2015 Health Information National Trends Survey (HINTS), about 34% of U.S. adults over 45 years old used mHAs [37], while according to Accenture’s 2018 survey of 2,301 Americans aged over 18, 48% of them use mHAs. This is much higher than 16% in 2014 [45]. While this might reflect the degree of penetration of mobile technology by country, it is clear that increasing numbers of people use mHAs and mobile technology. Nevertheless, high proportions of mHA users does not necessarily imply that people are using all facets of their mobile applications properly. Indeed, almost half of the mHA users in our study responded incorrectly to questions about the functionality of Bluetooth or Wi-Fi, which may be essential knowledge for using mHAs reliably. In addition, 50% of the mHA users expressed difficulties in learning how to use mHAs.

In our study, self-confidence was significantly associated with mHA use. Technical confidence is positively associated with perceived ease of use, which can affect behavioral intention and actual behavior [22, 46–48]. In multinational studies, diabetes patients’ perceived ease of use was associated with intention to use mHAs to manage diabetes [49]. Another study of 278 Portuguese adults between 55 and 94 years of age also found that perceived ease of use was associated with intention to use mHAs [50]. Therefore, educational interventions for mHAs utilization are recommended to improve self-confidence as well as to provide skills and knowledge to use mHAs.

In terms of perceived benefits, study participants perceiving mHAs as useful for self-monitoring, obtaining tailored health information, or maintaining healthy lifestyles were more likely to use the apps than people who did not. This is consistent with the results of a meta-analysis of 35 articles published from 2008 to 2016, which concluded that perceived usefulness had a significant impact on mHA use [20]. Considering that perceived benefits can affect intention to use mHAs, it would be important to allow potential or current mHA users to experience the benefits of mobile health functionality firsthand. However, availability of applications–without supported learning–may be insufficient. About a third of the study participants have not used mHAs and more than one-third did not know about the benefits of mHAs despite this study being conducted at tertiary hospitals where several mHAs were provided to patients for free. Therefore, it is important to inform patients about the potential benefits of the mHAs as well as guiding patients on how to use them well. In particular, considering patients are highly influenced by their physicians, applying mHAs to routine care by physicians might be a good strategy for doing so.

In our study, perceived barriers were not associated with mHA use. Study participants seemed not to be aware of the barriers compared to benefits. According to previous studies, major barriers to mHA use were cost, lack of familiarity with technology, and decreased visual and cognitive function [24, 33]. Nevertheless, only one-fifth and one-tenth of study participants perceived text size or costs as barriers to mHA use, respectively. These technical or economical barriers seemed to be resolved by improvements to the general IT environment. Most devices and services now provide assistive technology, and free public Wi-Fi is common. Still, people report needing technical assistance and experience difficulties in usage even when sensory and cost barriers are removed. Indeed, about half of our study participants, even mhealth app users, felt a lack of support for learning how to use mHAs. Thus, institutions and health professionals need to consider how to support patients in using mHAs in clinical settings.

Unfortunately, we were not able to include all factors associated with mHAs, such as hedonic motivation. Hedonic motivation, which is ‘joy of use,’ is an important predictor of technology adoption [50, 51]. A previous study of an older population (55–94 years) found that joy of use was associated with mHA utilization [50]. However, the effect of the factor was somewhat controversial depending on the type of digital health services or study setting [51, 52]. A qualitative study of mHAs found that participants aged 18 to 65 did not differentiate between hedonic motivation and usefulness [53]. Another qualitative study found that adults considered hedonic motivation to be more important for children or the younger generation than for themselves [54]. A comparison study conducted in Poland found that the adults aged 60 and over are less likely to experience hedonic motivation than the younger generation [55].

In addition, we did not include inter-personal factors (e.g. social influence) which might have impacted mHA use. According to previous literature, the subjective norm was not a significant factor in mHA adoption for older population. From this, we can infer that social influence by others’ beliefs or intentions would be similarly insignificant [34]. However, social supports such as physician recommendation or mHealth-related education at clinics would be associated with mHA use. Further study is necessary to examine all intra- or inter-personal factors associated with mHA use.

Our study has several limitations. First, this is a cross-sectional study and the associations between mHAs use and self-confidence and perceived benefits could be interchangeable. Second, people participated in this study who were interested in the topic, and the relatively high proportion of mHA users might be due to population selection. However, our results are similar to those of similar studies in other countries. Third, while our use of a convenience sampling method created a study population of relatively few older (aged 70+) participants, this is consistent with actual age-group distribution data presented in the 2018 Korea Census report [56]. Despite this similarity, it may still be difficult to generalize our findings for the broader over-70 population. Lastly, because this study was conducted at a tertiary hospital where mHealth technology is available in Seoul, Korea, the results of this study might not be generalizable to populations at other setting or in different countries. Korea is one of the most advanced countries in terms of Internet access and digital technology. In 2017, 91% of people aged over 50 own smartphones in Korea, which is the highest rate worldwide, and free public Wi-Fi is common across the country [57]. However, this is representative of a global trend. In fact, over 70% of the global population uses smartphones, and smartphone ownership is a major driving factor of mHA market growth [58]. Additionally, the global mHA market is expected to reach up to US$102.35 billion by 2023 [59].

In conclusion, mHA use among older adults is becoming more common globally, chronological age is becoming an insufficient predictor to explain mHA use in an aging society. However, there are still people unable to use mHAs properly because of lack of technical experience or knowledge. Thus, it needs to understand further the psychosocial or behavioral factors associated with the disparity within them related to mHealth, taking into account both homogeneity and heterogeneity between age groups. Our findings suggest that improving their self-confidence and informing people of the substantial benefits of mHAs would be a good solution to facilitate the use of mHAs, especially in the context of managing and controlling NCDs. In particular, implementing mhealth interventions based on such a strategy would help ensure better health outcomes in this era of digital therapeutics.

## Supporting information

S1 File A sample questionnaire for the survey. (PDF)

## Abbreviations

mHAs: mobile health applications
NCDs: non-communicable diseases
IT: information technology
