# Supplementary material for: The difference in knowledge and attitudes of using mobile health applications between actual user and non-user among adults aged 50 and older
Source: PLoS One. 2020 Oct 27;15(10):e0241350. doi: 10.1371/journal.pone.0241350 (PMC7591083; doi:10.1371/journal.pone.0241350)
Supplement: S1 File — (PDF) [file pone.0241350.s001.pdf]

Q1) Please choose all services you have used with a smartphone in the past 1 year and check (☑) them.

- ☐ 1. **Search for information** (Naver, Daum, etc.)
- ☐ 2. **Instant messaging** (KakaoTalk, LINE, etc.)
- ☐ 3. **Social media** (Naver Band, Kakao Story, Facebook, etc.)
- ☐ 4. **Public transit information** (Metro, bus app, etc.)
- ☐ 5. **Game** (Friends Popcorn, Go Stop, Anipang, etc.)
- ☐ 6. **Health** (Hospital portal app, Samsung Health, Noom Diet, Menstrual Calendar, etc.)
- ☐ 7. **E-mail** (Naver Mail, Daum Mail, etc.)
- ☐ 8. **Location-based service** (Kakao Navi, T Map, Naver Map, Daum Map, etc.)
- ☐ 9. **Financial transactions** (Online banking, transfer, securities transactions, etc.)
- ☐ 10. **Music and movies** (YouTube, Melon, Genie, etc.)
- ☐ 11. **Shopping and food ordering** (Baemin, home shopping apps, etc.)
- ☐ 12. **Other** ( )
- ☐ 13. **None**

Q2) Please check (☑) the right answer to each of the questions below about the black horizontal bar shown in the below image.

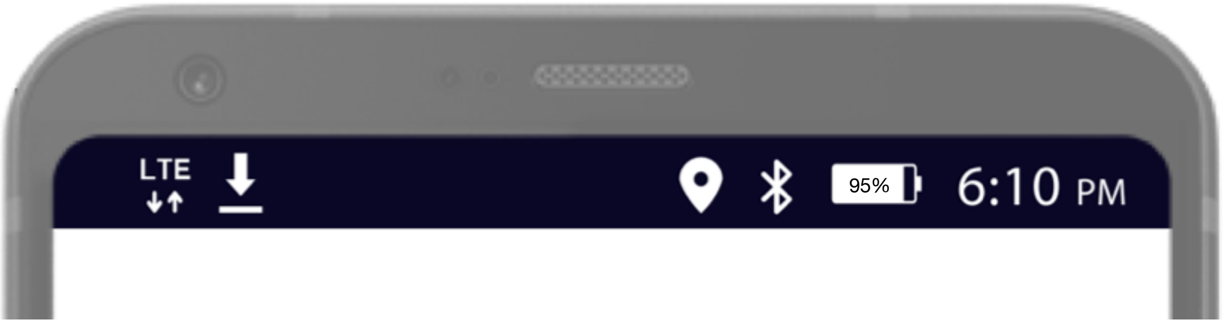

a.	The above smartphone can identify the user's current location.	1 Yes	2 No	0 I don't know
b.	The above smartphone is connected to WiFi (wireless internet).	1 Yes	2 No	0 I don't know
c.	The above smartphone can wirelessly connect to other electronic devices.	1 Yes	2 No	0 I don't know

Q3) Please read the following statements expressing personal beliefs about using mobile healthcare (mHealth app) and check (☑) the area that most closely represents your thoughts.

	Item	**Strongly Disagree**	**Disagree**	**Agree**	**Strongly Agree**
a.	If I learn, I can manage my health well with a smartphone.	1	2	3	4
b.	I am confident with using a mHealth apps well with a smartphone	1	2	3	4
c.	I am confident with using a smartphone well.	1	2	3	4

Q4) Please read the below <u>descriptions about barriers associated with using mobile healthcare (mHealth app)</u> and check (☑) the area you agree to.

	Item	Strongly Disagree	Disagree	Agree	Strongly Agree
a.	I think it will cost a lot of money to manage my health with a smartphone.	1	2	3	4
b.	I am concerned about privacy violation while managing my health with a smartphone.	1	2	3	4
c.	There is no person who can teach me how to manage my health with a smartphone.	1	2	3	4
d.	There is no place where I can learn how to manage my health with a smartphone.	1	2	3	4
e.	The text on the smartphone screen is too small to read.	1	2	3	4
f.	Even though using a smartphone for a while, my eyes easily feel tired.	1	2	3	4
g.	I don't know that mHealth app will really help me.	1	2	3	4
h.	I think using mHealth app will grow only the anxiety about my health.	1	2	3	4

Q5) Please read the following statements about <u>benefits of mobile healthcare (mHealth app)</u> and check (☑) the area that closely represents your opinion.

	Item	Strongly Disagree	Disagree	Agree	Strongly Agree
a.	The mhealth app will help me to find the health information I need	1	2	3	4
b.	The mhealth app will allow me to record my health status in real time.	1	2	3	4
c.	The mhealth app will allow me to get a health consultation without having to visit a hospital.	1	2	3	4
d.	The mhealth app will allow me to check my health status anytime, anywhere.	1	2	3	4
e.	The mhealth app will deliver tailored information for my health at the right time.	1	2	3	4
f.	The mhealth app will help me to maintain healthy behavior.	1	2	3	4

**Sex**	☐Male ☐ Female

**Date of Birth**	☐☐☐☐ Year ☐☐ Month ☐☐ Day

Final Education	☐ Middle school or lower ☐ High school ☐ Undergraduate ☐ Graduate or higher

Area of Residence	☐ **Seoul** ☐ **Incheon** ☐ **Daejeon** ☐ **Daegu** ☐ **Gwangju** ☐ **Ulsan** ☐ **Busan** ☐ **Sejong** ☐ Gyeonggi ☐ Chungbuk ☐ Chungnam ☐ Gangwon ☐ Jeonbuk ☐ Jeonnam ☐ Gyeongbuk ☐ Gyeongnam ☐ Jeju
Employment and Occupation	**Are you currently employed (engaged in economic activities)? (including self-employment)** ☐ Yes ☐ No
Monthly Average Household Income	**What is the monthly average income of all the household members that you live with?** **(including income from pensions, interest, rent, etc.)** ☐ Under KRW 1 million ☐ KRW 1 million – 299 million ☐ KRW 3 million – 499 million ☐ KRW 5 million – 6.99 million ☐ KRW 7 million or above

Comorbidity	**Please check (☑) all of the disease you have been diagnosed with or received treatment for in the past one year.** ☐ Liver disease (e.g. chronic hepatitis, cirrhosis, etc.) ☐ Hyperlipidemia ☐ Hypertension ☐ Stroke ☐ Diabetes ☐ Arteriosclerosis ☐ Chronic obstructive pulmonary disease (COPD) ☐ Myocardial infarction ☐ Cancer ☐ Prostatomegaly ☐ Asthma ☐ Degenerative arthritis ☐ Angina ☐ Other conditions (details:) ☐ None
